# Core Activation Program and Selective Regional Responsiveness of Microglia in Aging and Parabiosis

**DOI:** 10.1002/alz70855_106438

**Published:** 2025-12-24

**Authors:** Huma Naz, Robert Palovics, Shinnosuke Yamada, Nannan Lu, Tony Wyss‐Coray, Qingyun Li, Guoyan Zhao

**Affiliations:** ^1^ Washington University, St. Louis, MO, USA; ^2^ Stanford University, Stanford, CA, USA; ^3^ Washington University School of Medicine, St. Louis, MO, USA; ^4^ Wu Tsai Neurosciences Institute, Stanford University, Stanford, CA, USA; ^5^ Washington University School of Medicine, St Louis, MO, USA

## Abstract

**Background:**

Aging is the primary risk factor for many neurodegenerative diseases and is associated with immune dysregulation in the brain whereas rejuvenation interventions can mediate beneficial effects. Microglia are considered as a major player in neurodegenerative disease development yet, the molecular changes underlying brain aging and rejuvenation remain poorly understood at the single cell level.

**Method:**

Using scRNA‐seq we investigated microglia heterogeneity and their transcriptomics changes during aging and the parabiosis mediated exposure of young and old blood across four different brain regions including cerebellum, cortex, hippocampus, and striatum.

**Result:**

We identified eight distinct microglial subpopulations shared across different ages, brain regions and treatment conditions. By comparing the expression of subpopulation‐specific signature genes, (e.g. homeostatic microglia, disease associated microglia, and interferon response microglia), we benchmarked the identified microglia subpopulation, seven of which were reproducible in an independent widely used aging dataset. We nominated combinatorial signaling codes governing different reactive microglial states and defined the core microglial activation gene program. Additionally, we uncovered shared and region‐specific aging signatures across the four brain regions and identified region‐specific differential gene expression associated with cellular senescence, aging, amyloid metabolism and response to unfolded proteins. We investigated the effects of old blood and young blood on microglia and revealed that parabiosis‐mediated accelerated aging and rejuvenation had distinct effects on gene expression in specific brain regions, with the cerebellum consistently emerging as the most sensitive region and the striatum as the least affected. Pathways involved in apoptotic signaling, inflammatory responses, glial cell activation, synaptic plasticity, and phagocytic activity were differentially impacted across brain regions in response to aged and young blood exposure. Notable, we identified a core microglia activation signature including 13 genes (*Fth1, B2m, Cd9, Fcer1g, Selplg* etc.) that were upregulated in aging, old blood exposure, and across all reactive microglial populations within all brain regions tested. This points to a context‐independent, conserved mechanism regulating microglial reactivity.

**Conclusion:**

This work highlights the regional variation in microglial subpopulation transcriptomic dynamics during aging and under rejuvenation conditions, providing insights into differential brain regional vulnerability and offering potential avenues for microglia‐targeted modulation of brain aging.

